# Dynamic yet well-defined organization of the FUS RGG3 dense phase

**DOI:** 10.1038/s42004-026-01974-z

**Published:** 2026-03-21

**Authors:** Anton A. Polyansky, Benjamin Frühbauer, Bojan Žagrović

**Affiliations:** 1 Max Perutz Labs, Vienna BioCenter, 1030 Vienna, Austria; 2 University of Vienna, Vienna, Austria; 3 Institute of Biochemistry, ETH Zurich, Zurich, Switzerland; 4 Bringing Materials to Life Initiative, ETH Zurich, Zurich, Switzerland

**Keywords:** Computational chemistry, Biophysical chemistry

## Abstract

Intrinsically disordered protein regions (IDRs) play a key role in the formation of biomolecular condensates, a ubiquitous mode of cellular compartmentalization, but the underlying microscopic details remain unclear. Here, microsecond-level molecular dynamics simulations and fractal formalism are employed to study at atomistic resolution a model dense phase composed of 24 copies of a C-terminal 73-residue arginine- and glycine-rich IDR (RGG3) of fused in sarcoma (FUS) protein in the absence of RNA. RGG3 displays a highly dynamic behavior in the dense phase with only a small configurational entropy loss and a minor slowdown in diffusion as compared to the dilute phase. Despite rapid mixing, short contact residence times and structurally heterogenous binding interfaces in the dense phase, RGG3 exhibits a distinct dynamic binding mode, with statistically defined interaction motifs and a robust multi-scale topology of self-associated protein clusters. An analysis of bound water suggests that solvent entropy may significantly contribute to the thermodynamics of condensate formation. Our results demonstrate how a well-defined organization of the disordered protein dense phase across scales emerges from highly heterogenous, transient interactions at the molecular level.

## Abstract (web summary)

Intrinsically disordered protein regions (IDRs) play a key role in the formation of biomolecular condensates, but details of the underlying mechanism at the microscopic level remain at the center of ongoing studies. Here, the authors use microsecond-level molecular dynamics simulations and fractal formalism to study a model dense phase composed of 24 copies of a C-terminal 73- residue arginine- and glycine-rich IDR (RGG3) of fused in sarcoma (FUS) in the absence of RNA with atomistic resolution, showing that RGG3 exhibits a distinct dynamic binding mode with statistically defined interaction motifs and a robust multi-scale topology of self-associated protein clusters.

## Introduction

Intrinsically disordered regions (IDRs) in proteins play a significant role in different cellular processes, including transcription, translation and signaling^1–5^. The contribution of IDRs to these processes is frequently connected with their involvement in the formation of non-membrane-bound biomolecular condensates^6–8^, such as P-bodies^9^, stress-granules^10^, nucleoli^11^, nuclear bodies^12^ and others^6,13–16^. Importantly, many proteins with large IDRs display well-defined cellular functions, localization, phase behavior and robust binding to multiple targets^17^, while lacking a stable 3D-structural organization. A major open question concerns the emergence of reproducible and robust biological function from the extremely dynamic, disordered behavior of the underlying molecular constituents. For example, it is not clear how a wild-type protein can reversibly form fluid condensates and then, after a single amino-acid substitution in its large IDR, alter its preference in the direction of forming toxic fibrils (see e.g., ref.^18^).

Revision of the structure-function paradigm in the case of disordered proteins brings the primary amino acid sequence to the front. Indeed, a sequence defines how disordered a given region is and, therefore, directly or indirectly impacts all other related functions. Computational approaches for analyzing IDR sequences have revealed that certain compositional biases, presence of linear motifs, and even a “grammar” can be linked with the level of protein disorderedness^19,20^, interaction preference^21–23^, susceptibility to post-translational modifications^24^, and potential for biomolecular condensate formation^25–27^. However, IDR sequences usually have low complexity and are only weakly evolutionarily conserved, which presents significant challenges to a mechanistic understanding of the sequence-function paradigm. These challenges become even harder if one considers the dynamic nature of IDRs. Indeed, the folding energy landscape of IDRs is relatively flat, i.e., disordered ensembles consist of many structurally dissimilar, but similarly favorable configurations^28^ that exchange rapidly^29^. Thus, sequence properties of IDRs define their configurational ensembles, which need to be carefully considered when trying to understand IDR function^17^. This pertains to all levels of organization since, in the case of IDRs, structural heterogeneity and disorder transcend length scales and are equally relevant when it comes to local motifs, complete polymers or polymer networks. Consequently, configurational entropy^28,30,31^ becomes a key parameter that shapes IDR interactions and phase behavior. Specifically, structural reorganization and ordering of IDRs’ ensembles, which results from binding to a partner^28^ or transitioning into a dense phase, may result in substantial entropic costs^32,33^.

Atomistic details of IDRs’ ensembles and the determinants of their intra- and intermolecular interactions can be efficiently studied via computational modeling, specifically molecular dynamics (MD) simulations, which can also be efficiently integrated with experiment^34^. In particular, MD simulations of IDRs at the all-atom resolution are a unique biophysical tool, since they provide detailed information about the instantaneous position of each atom over realistic, microsecond timescales and, thus, allow direct observation of IDR configurational ensembles and interactions and enable a quantitative assessment of configurational entropy. This has, in particular, been made possible by recent extensive efforts at optimizing the existing MD force fields to generate IDR ensembles in agreement with experimentally derived constraints^35–38^. Thus far, all-atom MD simulations have been used to model conformational behavior of IDRs, interactions between IDRs and other proteins^38–40^ or small molecules^41^, and even direct modeling of biomolecular condensate formation^42^ and organization^32,43–45^. All-atom MD simulations, although being restricted in accessible system sizes and timescales as compared to various coarse-grained models widely applied currently in IDR modeling^38^, are much more accurate than the latter in probing the IDR dynamics in different context.

A recent MD study has revealed an intriguingly high level of protein dynamics inside a model condensate consisting of two highly and oppositely charged IDRs^42^. The authors could show that the studied IDRs remain extremely dynamic in the high-density environment (~300 g l^−1^) of the model condensate, despite a rich network of intermolecular interactions. Moreover, intermolecular contacts were found to be short-lived, with the characteristic relaxation times on the ns scale. In the context of the sequence-to-function relationship, are there any well-defined interaction patterns, which can be mapped to the primary sequence and which shape the organization of the highly dense and dynamic dense phase? The latter aspect is important in order to understand the mechanistic aspects of IDR association and condensate formation. Specifically, sequence features define different relevant parameters, such as IDR compactness^46–49^, multivalency^50–53^, and recruitment of RNA and other proteins into condensates^54,55^, while these parameters, in turn, define the mechanism(s) of IDR association and the properties of the ensuing condensates, e.g., their viscoelasticity^56,57^ and topology^32,58^. According to a recent model based on phase separation coupled to percolation, IDRs form a continuum of clusters of different size, whereby at high enough protein concentration, clusters grow, merge and coalesce into microscopically visible condensates^32,59^. The distribution of cluster sizes, which are experimentally accessible by scattering techniques, such as DLS, can be shifted toward small nanoclusters or large condensates by varying environmental parameters, including protein concentration, temperature or buffer composition^60,61^. Moreover, a recently derived fractal model provides a quantitative link between the atomistic features of an IDRs and the spatial organization of the biomolecular condensate it forms^32^, suggesting that parameters, such as condensate topology and density, may be directly encoded in IDR sequence.

**Fig. 1:**
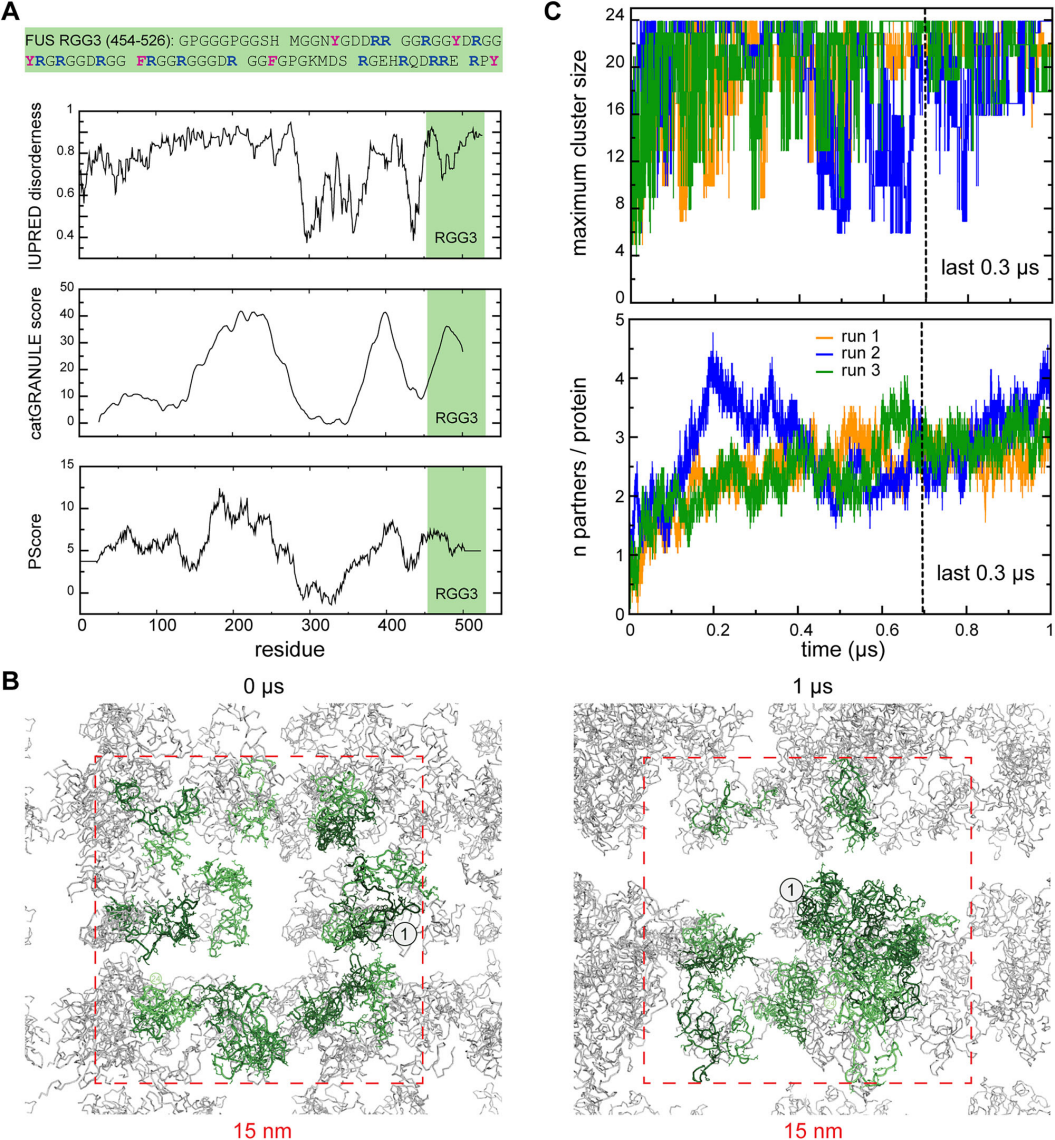
Self-association of the intrinsically disordered RGG3 in the dense phase. **A** Sequence of RGG3 with aromatic and arginine residues highlighted in magenta and blue, respectively. Panels below show per-residue prediction of RGG3 encoded propensity for intrinsic disorder (IUPRED) and LLPS (catGRANULE score and PScore). **B** Exemplary snapshots of a multi-copy system (the dense phase) at the beginning (0 µs) and the end of MD simulations (1 µs) with individual protein copies in the central simulation box shown in green and periodic images shown in gray. The simulation box is indicated with a dashed square. The protein copy 1 is indicated explicitly. **C** Time evolution of the largest detected protein cluster size (see “Methods” for details) and the average valency in the dense phase as calculated for 3 independent MD replicas of the multi-copy system, which are shown in orange (run1), green (run2), and blue (run3).

The RGG3 fragment (residues 454–526, Fig. 1A) of the human RNA-binding protein (RBP) FUS (fused in sarcoma) is a representative example of an RBP IDR that contributes to condensate formation and is involved in both protein-protein and RNA–protein interactions. FUS is found predominantly in the cell’s nucleus^62^, where it regulates transcription, RNA processing and DNA repair^63,64^. It is also enriched in neuronal cytosol^65^ and cytosolic ribonucleoprotein granules^66^, and can migrate to the cytosol upon DNA damage^67^. FUS forms condensates on its own, with both the N-terminal low-complexity (LC) and the C-terminal RGG regions being required for the full effect^68,69^. For example, removing RGG3 results in 4 times weaker condensate formation, even in the absence of RNA^70^. Importantly, RGG3 contains a number of charged residues that can drive condensate formation via long-range electrostatic interactions^13^. The abundant Arg and aromatic residues in FUS^71^ have also been suggested to contribute to condensate formation via short-range interactions, as also seen more generally^25,32,48,52,72–74^. Finally, RGG3 forms condensates in the presence of sub-stoichiometric amounts of MAPT RNA (1:50) at low salt, with Arg, Tyr and Phe residues being essential for the process^70^. It has been shown that both methylation of Arg residues in RGG3 and importin binding can abolish phase-separation of FUS^75^. More generally, Wake et al.^76^ have recently suggested that RGG domains may impact condensate formation of FUS also through homotypic (RGG–RGG) interactions, which have not yet been studied extensively.

To study how a defined and predictable organization of the IDR dense phase can emerge from highly disordered and dynamic interactions, microsecond all-atom MD simulations were employed to investigate the structure, dynamics, configurational entropy and architecture of intra- and inter-molecular interaction networks of the FUS RGG3 fragment and to compare those properties between simulations of a single copy and 24 copies of the protein in a box (Table 1). The former represents the protein in the dilute phase, while the latter is aimed to model the interior of a biomolecular condensate, i.e., the dense phase. The starting protein concentration of 89.2 g l^−1^ used here is by a factor of 2–5 lower than the known dense-phase concentrations of different IDR systems, including FUS itself^77^, FUS N-terminus^78^ or DDX4 N-terminus^79^, and it should allow one to observe the formation of the protein dense phase and study its properties. Note that if the starting concentration is above the actual dense-phase levels, then our simulations can be taken as the model of the local, high-density regions in the condensate. This may increase the protein valency and provide information on the local properties of higher-order clusters. The modeling framework used, which enables a multiscale description of the RGG3 condensate, has previously been applied to the disordered Lge1 fragment^32^ and RNA polymerase II C-terminal domain^45^. The results demonstrate that RGG3 displays a highly-dynamic and well-mixed behavior with structurally heterogenous binding interfaces and only a small configurational entropy loss upon transitioning to a dense, condensate environment, while at the same time exhibiting a characteristic dynamic self-interaction mode, with statistically defined interaction hotspots and a reproducible topology of self-associated protein clusters across length scales.

**Table 1:** Details of simulated systems

System	Box size	MD time, µs	Sampling step, ps	Proteins	# atoms				
					Protein	Water	Na^+^	Cl^−^	Total
Single copy 1	9 nm	1.0^a^	10	1	1010	94,972	44	51	96,077
Single copy 1 NVT	9 nm	0.05	10	1	1010	94,972	44	51	96,077
Single copy 2	9 nm	1.00	5	1	1010	95,004	44	51	96,109
Single copy 3	9 nm	1.00	5	1	1010	95,020	44	51	96,109
24 copies:									
Run 1	15 nm	1.00	2	24	24,240	407,076	203	371	431,890
Run 2	15 nm	1.00	2	24	24,240	406,692	203	371	431,506
Run 3	15 nm	1.00	2	24	24,240	406,692	203	371	431,506
Run 1 NVT	15 nm	0.05	10	24	24,240	407,076	203	371	431,890
Run 3 NVT	15 nm	0.05	10	24	24,240	406,692	203	371	431,506

^a^Total simulation time is 1.5 µs, for the final analysis 1 µs fragment used for the consistency.

## Results

According to IUPred2A^19^ analysis (Fig. 1A), the full FUS RGG3 sequence is predicted to have a high degree of disorder, which is supported by previous experimental evidence^80,81^. Furthermore, RGG3 (Fig. 1A) exhibits regularly spaced aromatic residues throughout, which are a known characteristic of proteins forming biocondensates^52^, as well as RGG repeats, which are prone to forming π–π interactions, contributing to condensation^26^. Indeed, the compositional bias of RGG3 is reflected in a high predicted propensity towards phase-separation according to catGRANULE^82^ and PSscore^26^ (Fig. 1A).

### RGG3 self-associates in the dense phase

The microsecond MD simulations reveal a tendency for the RGG3 fragment to self-associate in the dense phase (Fig. 1B). Specifically, the highly dynamic individual RGG3 chains form a complex network of intermolecular contacts (Fig. 1B), characterized by a rapid exchange of binding partners and the formation of protein clusters of different sizes (Supplementary Movie 1). The maximum cluster size increases with time in all three replicas (Fig. 1C) and settles in a relatively wide range (16–24 protein copies) over the last 0.3 µs of simulation time. Interestingly, the system is able to form only a transient percolating cluster, indicating that the contact probability is lower than it is required for a stable single percolating cluster with 24 interconnected copies and robust network transition. Specifically, the probability of proteins to form contacts with neighbors is related to the average valency of interactions, a measure of how many other copies a protein can coordinate. This parameter for RGG3 increases with time in all three multi-copy systems (Fig. 1C) and stabilizes around 3.3 ± 0.3 over the last 0.3 µs, with a relatively small deviation between the replicas (Table 2). Given the convergence in valency over this stretch, the last 0.3 µs of each of the three simulations were used for the analysis of RGG3 compactness and network topology.

**Table 2:** Summary table of observables derived from analysis of MD trajectories

Obervables	24 copies run 1	24 copies run 2	24 copies run 3	<24 copies>	Single copy 1	Single copy 2	Single copy 3	<Single copy>
Solvent accessible surface area (SASA), nm^2^	75.59	76.85	75.67	76.04	72.51	74.84	69.76	72.37
± St. dev.	4.91	2.80	4.94	4.31	4.67	7.22	6.37	6.52
SASA for all copies as one (per protein), nm^2^	62.37	62.42	62.71	62.50				
± St. dev.	2.39	2.38	2.52	2.43				
Relative exposure change (single to 24 copies)	−0.14	−0.14	−0.13	−0.14				
± St. dev.	0.03	0.03	0.03	0.03				
MIST configurational entropy (S_MIST_), a.u.	−5698.36	−5695.30	−5685.49	−5693.05	−5660.87	−5634.56	−5663.43	−5652.95
± St. dev.	23.91	29.21	24.65	26.25				15.98
∆S_MIST_ (single → dense), *R* units^a^	−45.41	−42.35	−32.54	−40.10				
± St. dev.	23.91	29.21	24.65	26.25				
Diffusion coefficient (*D*_t_^*pred*^), × 10^−6^ cm^2^ s^−1^	1.15	1.03	1.10	1.09	1.47	1.73	1.58	1.60
± St. dev.	0.13	0.15	0.12	0.14				0.13
Last 0.3 µs								
Radius of gyration (Rg), nm	1.82	1.91	1.89	1.87	1.71	1.80	1.40	1.64
± St. dev.	0.39	0.38	0.45	0.41	0.20	0.31	0.10	0.28
Valency (*n*)	3.13	3.45	3.32	3.30				
± St. dev.	0.20	0.41	0.21	0.32				
Volume fraction (*φ*)	0.44	0.38	0.42	0.41	0.47	0.45	0.82	0.60
± St. dev.	0.20	0.17	0.23	0.20	0.17	0.22	0.17	0.26
Fractal dimension (*d*_F_)	1.89	1.81	1.89	1.86				
± St. dev.	0.07	0.04	0.05	0.07				

^a^*R* is the universal gas constant.

### RGG3 dense phase is dynamic and structurally heterogeneous

**Fig. 2:**
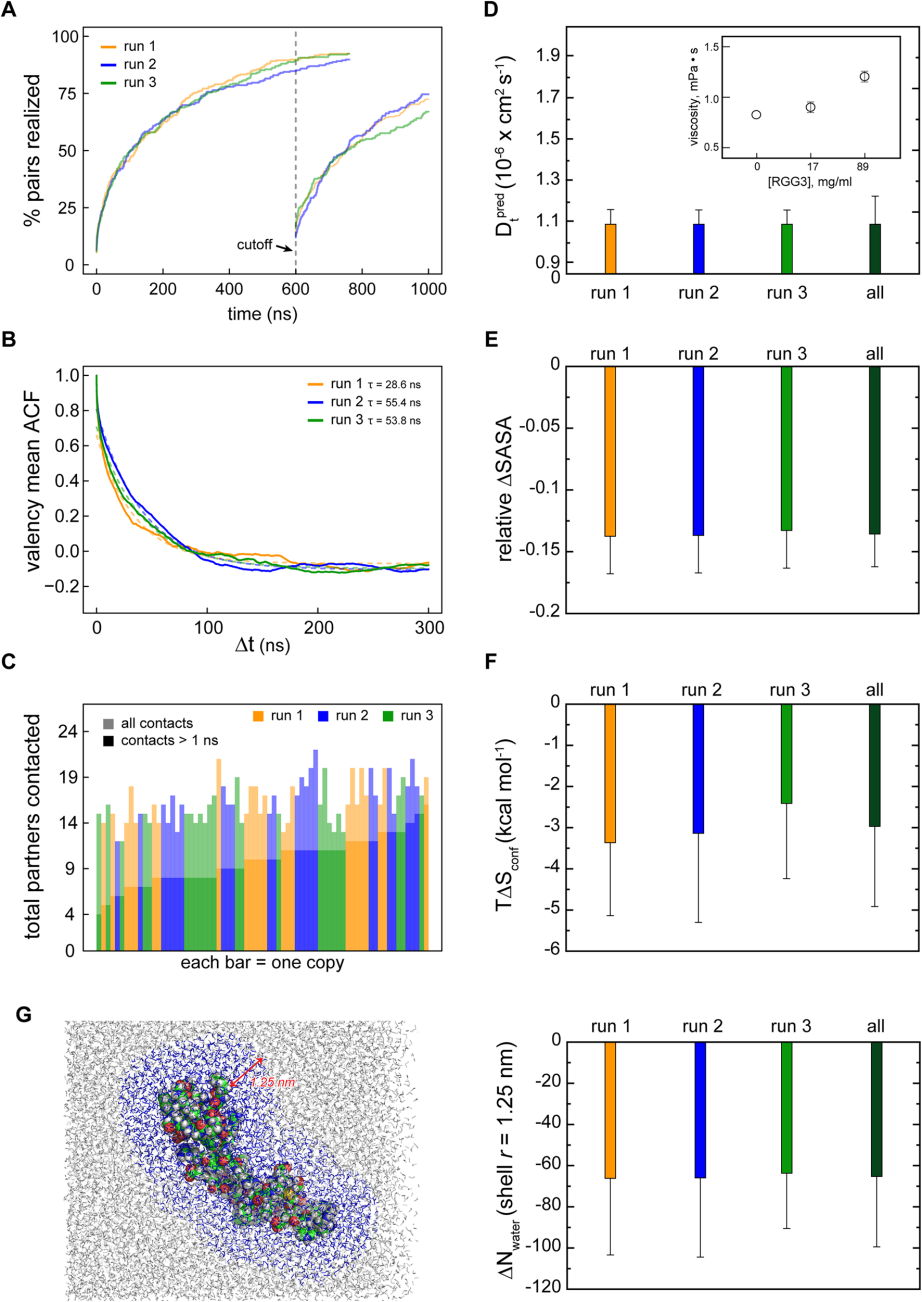
Dynamic and structurally heterogeneous nature of the RGG3 dense phase. **A** Fraction of all 276 (=24 × 24/2) possible pairwise intermolecular contacts that were established at least once after *t* = 0 µs or *t* = 0.7 µs in the three multi-copy system replicas. **B** Average autocorrelation function (ACF) of protein valencies as a function of time separation Δ*t* along each independent MD trajectory. Single exponential fits of ACF curves are shown with dashed curves, while the corresponding characteristic times of the fits are indicated in the legend. **C** The number of binding partners for each of the 72 individual protein chains over the last 0.3 µs of multi-copy simulations, with the contacts longer than 1 ns shown in darker colors. **D** MD-derived translational diffusion coefficients (see “Methods” for details) of RGG3 in the dense phase. The value was averaged between all 24 protein copies in the system. Error bars depict standard deviations. *Inset:* shear viscosity values obtained from the analysis of the pressure tensor autocorrelation functions (see “Methods” for details) and shown as a function of RGG3 mass concentration. The average values over different pressure tensor elements are shown together. Error bars depict standard deviations. **E** The average relative changes in the solvent accessible area (SASA) of RGG3 upon transitioning into the dense phase. Averaging was done for SASA differences in all possible combinations between three independent runs of single RGG3 and 24 protein copies in each complete 1 µs MD trajectory of the dense phase. Error bars depict standard deviations. **F** Average changes in the configurational entropy (Δ*S*_conf_) of RGG3 upon transitioning into the dense phase. Entropy values are given in energy units (*T*Δ*S*_conf_ at *T* = 310 K) and were obtained using complete 1 µs MD trajectories. Averaging was performed for entropy differences in all possible combinations between three independent runs of single RGG3 and 24 protein copies in each MD replica of the dense phase. Error bars depict standard deviations. **G** *(Left)* the bound water shell within the effective radius of 1.25 nm of the single RGG3 copy and *(right)* the average change in the number of bound water molecules in the 1.25-nm shell as compared between isolated proteins and the dense phase and estimated from RDFs that were obtained using complete 1 µs MD trajectories. The averaging was performed over the differences in the number of bound water molecules in all possible combinations between the three independent runs of single RGG3 and 24 protein copies in each MD replica of the dense phase. Protein is shown using sphere representation, while water molecules are shown with sticks, with the bound shell water molecules shown in blue. The size of the bound shell is indicated with a red arrow.

The instantaneous valency of RGG3 ranges between 0 and 9 binding partners per protein in all three multi-copy simulations (Supplementary Fig. 1A), reflecting a variety of bound configurations adopted by the protein in a crowded environment. Importantly, such interactions are characterized by a frequent partner exchange, which is well illustrated by the number of possible intermolecular pairs realized as function of time (Fig. 2A). In particular, over 90% of all possible pairs of individual RGG3 chains establish at least one direct contact in the course of 1-μs multi-copy simulations, while approximately 60% of all possible pairs contact each other at least once over the last 0.3 µs. The interaction dynamics can also be captured by the rate of exchange of binding partners via the valency autocorrelation function. Specifically, in all three multi-copy simulations, valency persists at a given level between 30 and 40 ns on average, with any memory of valency dissipating by ~150 ns (Fig. 2B). In fact, although the average valency is only ~3.3, each protein chain interacts directly with ~14 out of 23 possible different partners over the last 0.3 µs, whereby contacts with more than 50% of partners last on average shorter than 1 ns each (Fig. 2C). Clearly, contacts between different RGG3 copies form and dissolve rapidly and there is a high degree of mixing on the time scale of our simulations, creating a foundation for a detailed statistical analysis of interaction motifs (see below).

Highly dynamic interactions with frequent, nanosecond-level binding and unbinding suggest that RGG3 may diffuse rapidly in the crowded environment. A linear dependence of protein mean-square displacement (MSD) on time, indicative of an ideal diffusive behavior, was identified between 10 and 30 ns and used for deriving diffusion coefficients in the multi-copy systems (Supplementary Fig. 1B). Interestingly, the single-copy simulations exhibit no significant linear regions in the MSD vs. time curve. Fitting the MSD-curves in the range from 0 to 5 ns, as performed before^83^, still allowed estimation of the translational diffusion coefficient in those cases. Overall, both viscosity and diffusion coefficients derived from simulations (Table 2 and Fig. 2D) are in good agreement with the behavior expected from previous experimental and theoretical results. Specifically, our water model exhibits an apparent viscosity of 0.83 ± 0.01  mPa s, close to an experimental estimate of 0.69 mPa s^84^ (Fig. 2D and Supplementary Table 1), with the value increasing by ~10% and 50% for single-copy and multi-copy RGG3 systems, respectively. Furthermore, the translational diffusion coefficients of proteins in our simulations agree with previous simulation results^32,83^. Interestingly, the average diffusion coefficient of RGG3 in the single-molecule context (1.60 ± 0.13 × 10^−6^ cm^2^ s^−1^) reduces by only 32% in the dense phase (1.09 ± 0.14 × 10^−6^ cm^2^ s^−1^) (Table 2). This result indicates that the transient interactions with other protein partners and association into clusters slow down the diffusion of relatively short RGG3 only marginally, in contrast with the behavior of full-length FUS, for which a slowdown of several hundred-fold was experimentally observed^85,86^.

Formation of the protein network results also in a 14% reduction of protein solvent-accessible surface area (SASA) in the dense phase as compared to the single-copy context (Table 2). Specifically, the average SASA for RGG3 over all three multi-copy systems is 62.5 ± 2.4 nm^2^, with very small differences between the replicas, while this variation is a few times higher for single-copy replicas (Fig. 2E). The systematic reduction of SASA upon crowding is also reflected in the thermodynamics of RGG3 self-association. Thus, calculations show that the configurational entropy of disordered proteins systematically and reproducibly decreases upon crowding, with an average *T*Δ*S*_conf_ per molecule of −3.0 ± 2.2 kcal mol^−1^ (Fig. 2F). However, due to the very dynamic organization of the protein network, this effect is significantly smaller as compared to the Lge1 (1–80) fragment (*T*Δ*S*_conf_ = −6.6 ± 3.5 kcal mol^−1^), which was shown to robustly form condensates in vitro and stable percolating protein clusters in MD^32^. Interestingly, the reduction of the RGG3 configurational entropy upon crowding in all three multi-copy systems is linearly proportional to the relative reduction of its SASA (Supplementary Fig. 1D).

To assess how self-association of proteins affects the bound water shell organization, a radial distribution function (RDF) was calculated for the complete 1-µs trajectories of the dense-phase and single-copy (Supplementary Fig. 1E) systems. All estimates of the change in the number of water molecules between the dense and the single-copy systems were made for a water shell with the effective radius of 1.25 nm (Fig. 2G). This characteristic distance was defined through the analysis of a distance-dependent variation in the number of water molecules between the independent runs in the single-copy systems (see “Methods”) and agrees well with the prior estimate of the thickness of a bound water shell surrounding a protein of ~1 nm^87^. The thus defined water shells become depleted by ~65 molecules per protein chain when going from a single non-interacting copy to the dense-phase system (Fig. 2G). Interestingly, a similar depletion is observed when comparing the RDF for the dense-phase system between the first and the last ns of MD, while for the single-copy system the number of water molecules remains stable during the MD simulations (Supplementary Fig. 1F). The depletion of the bound water shell was also estimated from the absolute change in SASA, using a conversion factor of 0.15 nm^2^ of SASA per bound water molecule^88^, which gives very similar numbers for each independent run as compared to the direct count (Supplementary Fig. 1F). Assuming that the release of each water molecule corresponds to ~7 cal mol^−1^ K^-1^ ^89^, it is possible to estimate the upper bound of solvent entropy change due to the RGG3 self-assembly, which displays a negative linear relationship with the respective change in the conformational entropy over the simulated systems (Supplementary Fig. 1H). Here, the possible correlations between the released water molecule, which would reduce the effective entropy values, are neglected. Under this assumption, the water entropy increases significantly more in absolute value upon the formation of the dense phase as compared to the decrease in the conformational entropy per protein (~141 kcal mol^−1^ vs. ~−3 kcal mol^−1^).

### Interaction motifs in RGG3 can be statistically defined

**Fig. 3:**
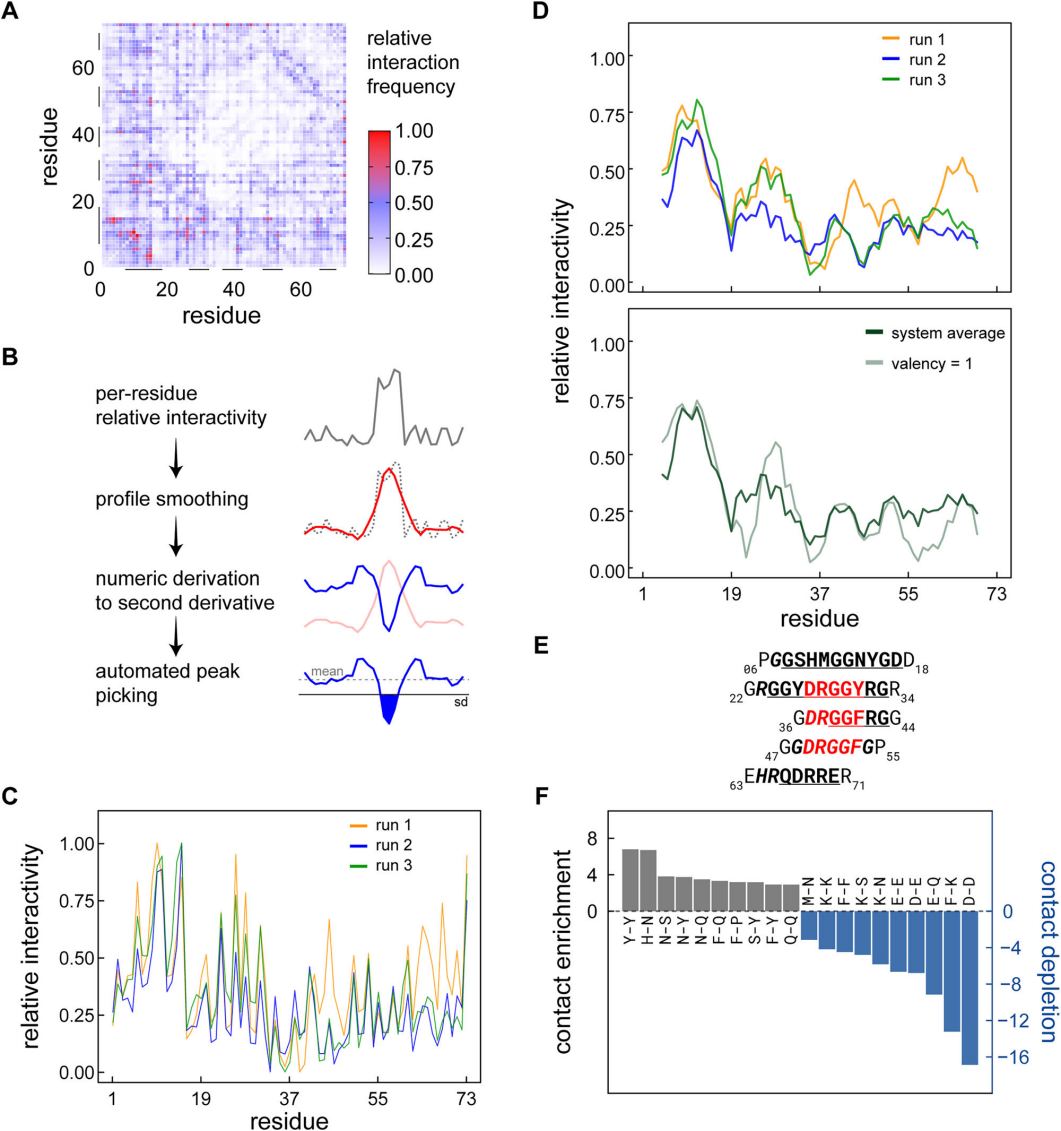
Statistically defined interaction motifs in RGG3. **A** Per-residue heatmap of intermolecular interactions. Bars highlight positions that are subsequently identified as significant drivers of intermolecular interactivity. **B** Overview of Savitzky–Golay filtering and peak-picking (see “Methods” for details). **C** Unfiltered (raw) relative residue interactivities (contact formation frequencies) of RGG3 in the dense phase. **D** Contact frequencies profiles of RGG3 for individual multi-copy simulation replicas and comparison of interactivity profiles at all valencies and at valency 1. **E** Alignment of interacting regions (stickers) in RGG3 as identified by Savitzky–Golay filtering for the combined set based on all three trajectories at all valencies: the stickers are shown either in bold and underlined (identified for all three smoothing window sizes) or bold and italic (identified for two out of three smoothing window sizes). The common DRGG(F/Y) motif (labeled in red) was identified in all three interaction hotspots located in the RGG-rich region of the protein. **F** The ten most enriched and depleted types of pairwise intermolecular contacts involving molecules with a valency of 1 in the RGG3 dense phase.

To study which regions in RGG3 are involved in the above interactions, a detailed analysis of all pairwise intermolecular contacts was performed (Fig. 3A and Supplementary Fig. 2A–F). For better statistics, the averaging interval of MD trajectories was extended to 0.4 µs in the contact analysis. Consistent with their high dynamics, RGG3 chains interact with each other throughout the entire sequence, but to a variable degree. To allow identification of these regions, the raw profile of contact formation frequencies as a function of sequence position (Fig. 3B) was processed via the Savitzky–Golay filter (Fig. 3C and Supplementary Fig. 2B, C). Specifically, the N-terminus of RGG3 (residues 1–19) exhibits pronounced interactivity in all three replicas, with several additional regions also featuring. Remarkably, despite high structural heterogeneity of simulated systems, an alignment of individual interactivity profiles from different replicas (Fig. 3D, top) results in a good overall agreement, with pairwise Pearson Rs around 0.8 or greater. This is indicative of a strong convergence when it comes to interaction propensities. An exception can be seen in the third peak, which is located around residue 45 in run 1, while being shifted noticeably to the left in runs 2 and 3. Notably, a sequence interaction profile with the cleanest separation between interaction hotspots is obtained if one focuses just on conformers with valency = 1 i.e., chains that interact with one other partner only at a given time point (Fig. 3D, bottom). These conformers may inform on the residues involved in the early contact formation. Finally, one can use the Savitzky–Golay filter to identify sequence regions that mediate the formation of intermolecular contacts in an unbiased way. Using this approach, a repeating DRGG(F/Y) motif was identified within the RGG-rich region as a driver of intermolecular contact formation in RGG3 (Fig. 3E and Supplementary Fig. 2D). Interestingly, the DRGGF motif closest to the N-terminus is determined with the highest confidence, while the subsequent motifs show a continuously decreasing interactivity. A possible explanation for this can be found in the residues surrounding each motif: the first is flanked by the highly interactive Tyr and Arg, while the third motif is surrounded by Gly residues, which likely cannot stabilize contacts to the same degree. On the other hand, recent findings show that, due to its impact on local flexibility, glycine can increase the sampling of neighboring residues and, in this way, indirectly stimulate contact formation^76^.

The 20 most frequent types of residue-residue contacts between different copies of RGG3 are shown in Supplementary Fig. 2E. Many of these involve residues that are abundant in the protein’s sequence, most notably Gly. To account for this, the 10 most enriched and the 10 most depleted pairs of residues among the observed contacts were identified and normalized by their abundance in the RGG3 sequence (Fig. 3F and see Supplementary Fig. 2F for the statistics without filtering by valency). The contacts involving Tyr are significantly enriched in the multi-copy system, together with the contacts that are stabilized by hydrogen bonding, such as those involving Asn. Depleted contacts, on the other hand, generally tend to involve polar and charged residues. Notably, the depletion of Phe-Lys interactions is consistent with previous findings in other proteins^90^. Finally, both the enriched and the depleted contacts include residues of different size. This is important as amino acids with larger side chains would be more likely to contact each other by accident in a crowded environment. Thus, the relationship between residue size in terms of surface area and per-residue intermolecular interactivity were analyzed and compared for the whole system and for contacts at valency = 1 only (Supplementary Fig. 2A). The correlation between residue size and interactivity in the full system (*r* = 0.55) appears to be guided by the residues on the two ends of the size spectrum, and shows outliers among the medium-sized residues. At valency 1, however, the correlation largely disappears (*r* = 0.25), suggesting that the interactions are established more specifically in that case.

### RGG3 interaction motifs are intrinsically disordered and exchange rapidly

**Fig. 4:**
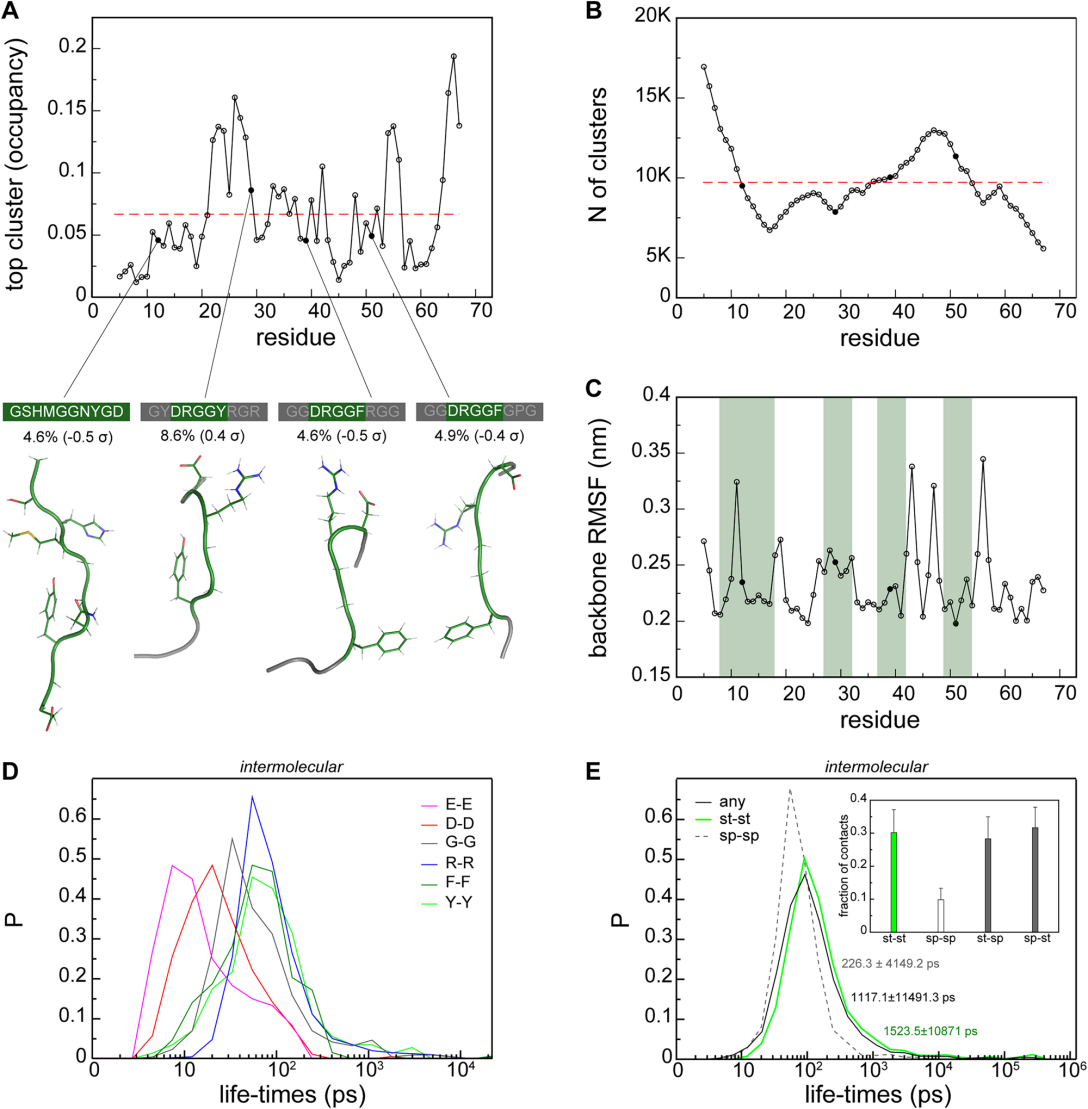
Disordered nature and dynamics of RGG3 interaction motifs. **A** Relative occupancy of the top conformational clusters of 10-residue fragments in RGG3. The average occupancy of top clusters is indicated with a dashed line, while central structures of top conformational clusters for the identified interaction motifs are shown below. Relative occupancies for the top clusters of the interaction motifs (in %) and deviation from the baseline (in standard deviations) are given below the motif sequences. For short interaction motifs, sequence neighbors within 10-mer fragments are shown with gray color. **B** Total number of delineated conformational clusters for each 10-mer fragment. The average is indicated with a dashed line. **C** Average per-residue root-mean-squared fluctuations (RMSF), using a sliding window of 10 residues. Positions of interaction motifs are highlighted in green. Life-times of homotypic contacts (**D**), contacts between interaction motifs (stickers) and their spacers (**E**) in the RGG3 dense phase. The distributions comprise all individual values of lifetimes for selected residual contact types obtained over the last 0.4 µs of the three independent runs of 24 protein copies. *Inset:* average fractions of all contacts distributed between the sticker and spacers regions. Error bars depict standard deviations.

In line with the predicted disorder of RGG3, structural clustering of the protein as a whole showed a marked absence of any conformational preference. However, it is possible that the interaction regions adopt locally well-defined conformations upon binding. Therefore, RMSD-based structural clustering was performed on consecutive 10-residue fragments along the RGG3 sequence (see “Methods”) to identify the most populated conformational cluster for each (Fig. 4A). The most highly occupied clusters, i.e., the most structurally well-defined regions, are located at the protein’s C-terminus, with an occupancy rate approaching 20%. However, the top conformational clusters that contain the identified N-terminal interaction hotspots or DRGG(F/Y) motifs exhibit mostly below-average occupancies, and a clear conformational similarity between the top clusters of the motifs is absent. Curiously, the weakly interacting regions i.e., spacers, appear to be conformationally better defined i.e., exhibit increased occupancies of the top conformational clusters (Fig. 4A). The total number of clusters in individual 10-mer stretches was also analyzed (Fig. 4B). The high number of clusters at the N-terminus and the low number of clusters at the C-terminus are consistent with the respectively low and high occupancies of the top clusters at these positions. The interaction motifs also overlap with the positions of highly fluctuating sequence regions (RMSF peaks, Fig. 4C), especially for the first two peaks. Interestingly, RMSF profiles generated for the combined MD trajectories (see “Methods”) represent a characteristic dynamic fingerprint of RGG3 in the dense phase, where the position of the regions with high conformational heterogeneity are rather independent from the size of the applied sliding window (not shown). Altogether, the systematically high number of local conformational clusters along the entire RGG3 sequence suggests that binding events that involve both high- and low-interacting regions, or stickers and spacers, respectively, are structurally heterogeneous and devoid of well-defined configuration, reminiscent of what has been termed a “fuzzy complex” ^91^.

To assess the characteristic timescale of interactions for the discussed motifs (Fig. 3E), time-resolved contact statistics were systematically analyzed (see “Methods”). The average lifetimes of homotypic contacts range from 10 to 100 ps, depending on the type, with individual values ranging anywhere between a few ps and ~10 ns (Fig. 4D). This agrees well with previous MD results^42^. Interestingly, anionic homotypic contacts exchange the fastest (average life-times of ~10 ps), while those involving Arg, Phe, and Tyr, which are prone to π–π interactions, exchange more slowly (average life-times of ~100 ps). Altogether, due to the compositional bias in the RGG3 sequence, the distribution of life-times covering all types of protein contacts at once are on average distributed similarly to the latter (Fig. 4E). Interestingly, spacer-spacer contacts exchange almost an order of magnitude faster than do sticker-sticker contacts (Fig. 4E). The clear difference between contact dynamics in sticker and spacer regions can be also seen at the level of individual fragments (Supplementary Fig. 2G). Interestingly, both stickers and spacers display wider distributions of lifetimes at the level of intramolecular interactions (Supplementary Fig. 2H) and generally exchange more slowly as compared to the intermolecular context. Overall, sticker-sticker interactions contribute ~30%, while spacer-spacer interactions contribute only ~10% of all observed contacts (Fig. 4E, inset). At the same time, stickers and spacers are also prone to establishing mutual interactions, accounting for up-to 60% of all intermolecular interactions (Fig. 4E, inset). According to this analysis, only the spacer–spacer interactions are prominently depleted in the dense-phase RGG3 system. The latter trend is to large extent preserved at the intramolecular level of interactions, with a slight increase in sticker-sticker contacts and a slight reduction in sticker-spacer contacts (Supplementary Fig. 2H, inset).

### RGG3 clusters exhibit robust scaling

**Fig. 5:**
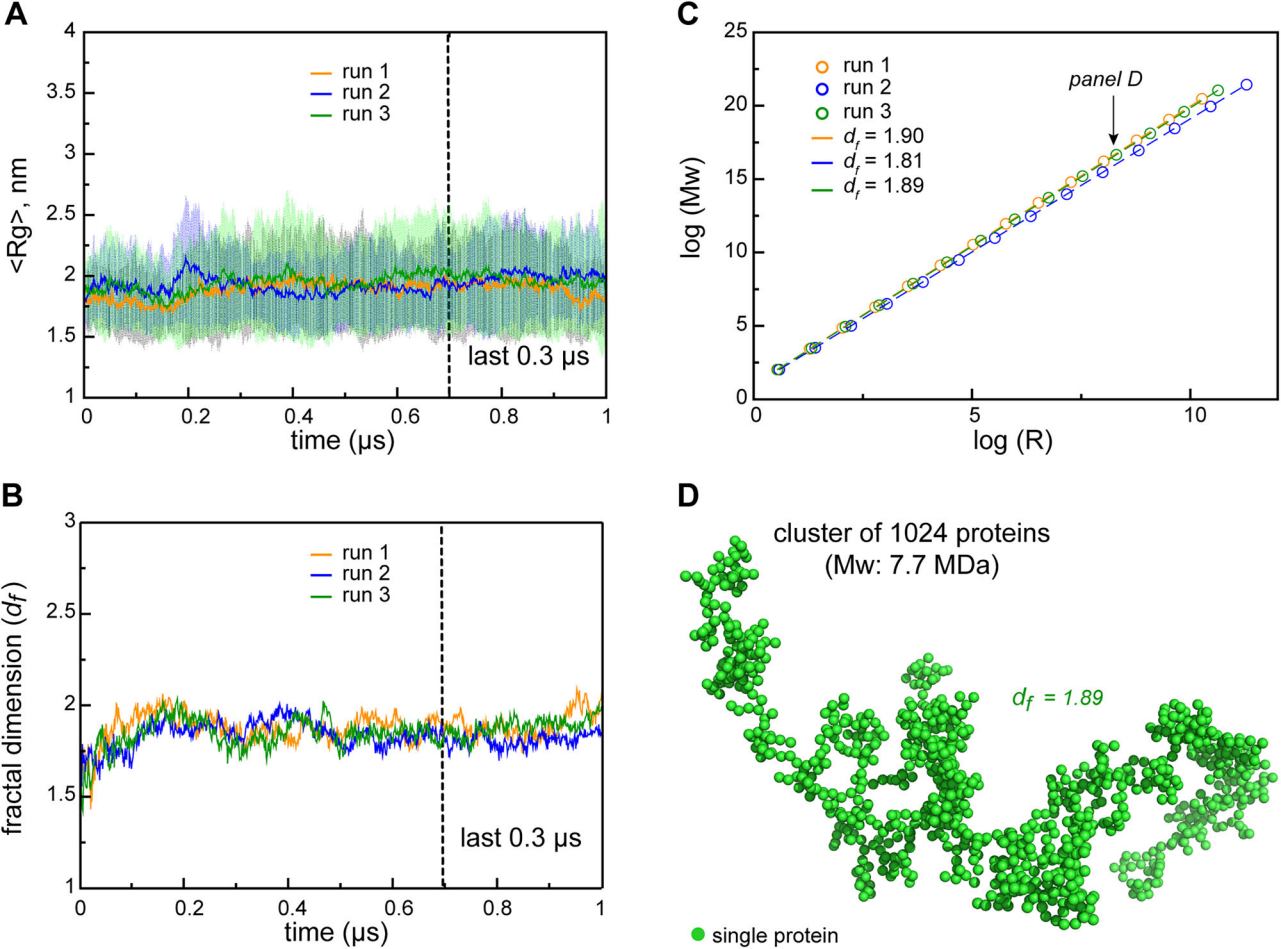
Robust scaling of RGG3 clusters. **A** Time evolution of the average radius of gyration (*R*_g_) in the RGG3 dense phase. The average values over all 24 copies are shown together with the standard errors of the mean. **B** Time evolution of the fractal dimension (*d*_F_) in the RGG3 dense phase. The fractal dimension is calculated using average *R*_g_ and valency values (Eqs. 11–13 and see “Methods”) between 24 protein copies at each time step of MD trajectories. **C** Power-law dependence between mass and size of RGG3 clusters at different iterations of the fractal model (see “Methods” for details) with the applied valency and compactness corresponding to their average values over the 24 simulated protein copies and the last 0.3 µs of each MD replica of the dense phase. Dashed lines show linear regression for the log *R* vs. log *M*_w_ plot with the corresponding *d*_F_ values indicated in the legend. **D** A representative coarse-grained 1024 particle cluster obtained by the FracVAL algorithm (see “Methods”). The cluster was reconstructed using the *d*_F_ and the average *R*_g_ value over the last 0.3 µs of the dense-phase MD simulations (run2).

To probe the topological organization of RGG3 protein clusters across different length scales and translate the observations obtained for the relatively small simulated systems to the sub-micrometer scale, the previously developed fractal model^32^ was employed (see “Methods”). According to the model, protein compactness and interaction valency define the spatial organization, or “dimensionality” of the self-associating clusters, across different length scales. Specifically, the fractal dimension (*d*_F_) of such protein clusters can be directly calculated from the values of the interaction valency (*n*) and the corresponding compactness of a protein (*ϕ*) using Eq. 11 (see “Methods”). Overall, RGG3 in single-copy simulations samples relatively compact conformations, with an average <*R*_g_*>* of 1.64 ± 0.28 nm for all replicas over the last 0.3 µs of MD (Supplementary Fig. 3A and Table 2). This is ~30% smaller than what would be expected for a random-coil peptide of this size according to a simple scaling law^92^. The observed compactness of the single-copy RGG3 indicates that intramolecular interactions shape the configuration of the protein to a significant degree. This value increases by ~14% in the crowded environment to 1.87 ± 0.41 nm, as averaged over all copies in all replicas (Supplementary Fig. 3B and Table 2). Thus, the dense phase promotes spatial extension of the RGG3 conformations due to intermolecular protein-protein interactions and intercalation in the emerging protein network. Interestingly, the average *R*_g_ in the multi-copy system displays a stable behavior along the most part of the MD trajectories in all three replicas (Fig. 5A), in contrast to the single-copy simulations, where fluctuations in *R*_g_ value are much higher (Supplementary Fig. 3C). Thus, the compactness of RGG3 is robustly defined in the crowded environment. This is reflected in the fact that the fractal dimension *d*_F_ exhibits a well-defined, nearly identical value for the three multi-copy replicas (Fig. 5B), with an average value of 1.86 ± 0.07 over the last 0.3 µs (Table 2). Therefore, while being very dynamic, the RGG3 protein network is expected to exhibit a stable topological organization across scales, with the fractal dimension below 2. This is consistent with the network exhibiting a low-dimensionality with a relatively loose organization. Such architecture, as predicted by the fractal model, should be valid for clusters of any size: indeed, the scaling between cluster *M*_w_ and its *R*_g_ remains constant and is very similar between all three multi-copy systems (Fig. 5C). Finally, reconstruction of the 3D organization for a cluster of 1024 RGG3 proteins with *M*_w_ of 7.7 MDa clearly demonstrates the relatively loose organization of the protein network (Fig. 5D) and a very similar large-scale architecture for the three independent MD replicas of the dense-phase system (Supplementary Fig. 3D).

## Discussion

A detailed characterization of the structure, dynamics and interactions of biomolecular condensates at an atomistic scale is an open challenge of both fundamental and applied significance. Here, systematic all-atom MD simulations were employed to provide a detailed view of the dense phase of a biologically relevant IDR fragment, gaining insight into what the driving forces behind phase-separation involving IDRs may be. The simulated RGG3 system is characterized by rapid mixing and a dynamic exchange of intermolecular contacts, with individual proteins remaining structurally disordered throughout simulations. Remarkably, however, the intermolecular contacts in the RGG3 dense phase exhibit robust, statistically well-defined features. Specifically, it was shown that a repeating motif, DRGG(F/Y) drives the formation of intermolecular contacts between different copies of RGG3 via interactions with the highly interactive N-terminal region. It is likely that the initial formation of contacts is guided by Arg residues, while the aromatic residues provide stability to the dynamic network of interactions, whereby characteristic life-times for aromatic contacts were shown to be within 10–10^3^ ps range.

Our results are in good agreement with the recent findings of Wake et al.^76^, who have expanded the molecular grammar of FUS phase separation by demonstrating the importance of interactions involving polar residues and arginine, including RGG–RGG interaction motifs. In general, the contacts that are enriched in our simulations correspond well with the observations from the Wake et al. study. Moreover, Rekhi et al.^93^ have used a set of designed protein sequences to show that Arg-Asp interactions are enriched in the protein dense phase, while the Asp-to-Asn mutant showed significantly lower condensate formation tendency, which is interesting as we identify Asp in the RGG3 interaction motifs. This may imply that electrostatic interactions may contribute to long-range attraction between chains.

Analysis of the present MD simulations using the fractal model of condensate architecture across scale shows that the simulated RGG3 system exhibits a remarkably robust and predictable behavior, with the three independent simulations of the dense phase resulting in almost identical values of the fractal dimension *d*_F_ and, consequently, almost identical scaling behavior (Fig. 5C, D, and Supplementary Fig. 3D). This entirely reflects the fact that the interaction valency and the compactness of individual RGG3 conformers in the dense phase exhibit well-converged, similar values across the different, independent simulations. More generally, this also illustrates a major feature and advantage of the fractal organization of the protein-dense phase: in such an organization, the large-scale architecture of the complete network is a direct consequence of the intrinsic features of individual chains it consists of, i.e., their valency and compactness, which in turn are encodable in protein sequence. Finally, the fractal nature of the protein-dense phase implies formation of clusters at different length scales, in agreement with the observed heterogeneous distribution of clusters in subsaturated solutions of the FUS-EWSR1-TAF15 family of proteins^94^.

Recently, it was proposed that the regular spacing of aromatic stickers in phase-separating IDPs weakens the strong interaction between aromatics through the favorable solvation of the intervening spacers and, in this way, optimizes the balance between desirable phase separation and undesirable aggregation^8,52^. The present results suggest that, additionally, polar residues participate significantly in contact formation and that the free-energetic difference between contacts involving sticker regions and those involving spacer regions may only be minor. Thus, the contacts involving sticker and spacers regions make up to 60% of all intermolecular contacts, while exclusively sticker-sticker contacts on average do not exceed 40% (Fig. 4E, inset). At the same time, exclusively spacer-spacer contacts contribute only ~10% of all contacts on average and exchange contacts substantially faster in the intermolecular context as compared to sticker-involving contacts (Fig. 4E). Thus, spacers in the dense phase are defined statistically as sequence regions that avoid interactions with themselves, but can still be engaged in establishing the intermolecular network by interacting with the stickers. This provides a counterpoint to the canonical sticker/spacer picture, in which spacer interactions are strongly disfavored. Rather, it is likely that all parts of a typical IDR participate in interaction, but with a continuum of interaction strengths and residence times. However, it is also possible that the present simulations capture primarily the early events in condensate formation, and that the interactions between stickers get stronger as condensates mature. This caveat also pertains to all structural and dynamical features of condensates analyzed herein.

A number of mutations that are commonly identified in FUS-related pathologies cluster in its C-terminally located NLS. Interestingly, the NLS contains two motifs identified by our analysis, _49_DRGGF_53_ and _66_QDRRE_70_. What is more, pathological mutations in these regions or their immediately flanking residues (G54D, R68C, R68G, R68H, or R69G) invariably involve perturbation of charge states. It is possible that these mutations affect intermolecular contact formation in FUS condensates by modulating electrostatic interactions and exert their deleterious effect in this way. Future mutagenesis work could provide a test of this hypothesis.

Here, it is important to emphasize that RGG3 was shown to phase separate in the presence of sub-stoichiometric amounts of RNA (1:50) only and, with nucleotides or RNA absent, our simulations may not provide a complete picture of how RGG3’s residues drive phase separation. In fact, it is possible that the primary function of the RGG3 region of FUS is to interact with RNA and increase local RNA enrichment. However, there are multiple lines of evidence suggesting that protein-protein interactions, as studied herein, may play a significant role in the process, including the high intrinsic propensity of the RGG3 sequence for phase separation as captured by different algorithms (Fig. 1A) and the central role of the fragment in the phase separation of FUS with, but also without RNA^70^. Our present work should provide a foundation for performing and analyzing similar simulations in the presence of RNA to study its impact on the network topology, valency and the nature of the relevant interaction motifs. In fact, by informing on the intrinsic propensity of the RGG3 polypeptide for self-interactions, the present analysis should provide a relevant baseline for such assessment of the impact of RNA. The RGG/RG motifs^76^, in particular, are expected to shift, at least in part, from participating in protein-protein interactions to establishing contacts with RNA. Finally, future work should shed light on how trace amounts of RNA may affect the network topology by influencing the compactness and valency of the constituent proteins.

An accurate analysis of IDRs via MD simulations poses significant challenges when it comes to both the force fields and the degree of sampling required. The choice of the Amber99SB-ILDN force field^95^ in combination with the TIP4P-D water model^36^ ensures that the simulated trajectories capture well the dynamic and disordered nature of RGG3. Such a setup, and in particular the usage of TIP4P-D to increase the dispersion interactions as compared to the TIP3P model, has been shown to yield accurate modeling of IDPs in water^35,96,97^. It was also used recently in our modeling of the dense phase of an Lge1-80 IDP, in good agreement with experiment^32^. On the other hand, some protein-protein interactions are still difficult to capture even by the latest force fields^36^, including π−π interactions^26^, which are thought to be a key driver of IDR phase separation. In particular, the strong enrichment of aromatic interactions seen in RGG3 may, to some extent, be due to a force-field bias, which could be magnified in a crowded environment, especially at high valency. On the other hand, our simulations also clearly point to polar residues as being involved in establishing early, specific contacts between individual RGG3 molecules. Such interactions may guide molecules to bind to specific sites, while a different subset of residues is responsible for contact stabilization.

The second challenge in simulating condensates is posed by a high degree of sampling needed to describe their structural and dynamic heterogeneity. The decision to assign almost 2 µs of MD simulation time to equilibration and combine three independent trajectories into a joint ensemble was taken precisely in order to adequately capture the statistical trends behind the behavior of the RGG3 condensate at the molecular level. Importantly, in each of the trajectories, RGG3 was simulated in multiple copies, greatly improving the degree of conformational sampling: altogether, the simulations included a total of 72 independent copies of the protein, reaching a combined total of ~30 µs of sampling at the level of individual chains in the converged state. Additionally, rapid partner exchange and transient formation of intermolecular contacts have enabled a statistically robust characterization of relative interaction propensities. Finally, there is a close agreement of the three independent trajectories in terms of the average number of binding partners per protein and, most importantly, residue-based interactivity profiles. While it is not possible to discount a qualitatively different behavior of the condensate on a time scale beyond microseconds, such convergence strongly suggests that the obtained data accurately represents the local equilibrium behavior of RGG3.

Importantly, the use of all-atom MD simulations allows direct and accurate assessment of the configurational entropy change for RGG3 upon formation of the dense phase. Here, the decrease in the configurational entropy is almost two times smaller than in another recently studied IDR-Lge1 (1–80)—which undergoes robust LLPS in vitro^32^. Interestingly, the entropic penalty obtained for RGG3 is at the same time of a similar magnitude as that for Lge1 (1–80) Y2A mutant with impaired LLPS^32^. The latter suggests that IDRs showing robust LLPS may exhibit a certain threshold entropic penalty that facilitates condensate formation. An interesting question here concerns how the loss of configurational entropy in the condensed phase is thermodynamically compensated. If the protein configurational entropy decreases prominently upon condensation, it should at the same time increase for some other parts of the system. Specifically, the entropic penalty for RGG3 is linearly proportional to the relative reduction of its SASA and release of water molecules. Moreover, the entropy of the solvent increases proportionally to the reduction of protein configurational entropy and this effect can significantly contribute to protein condensation in the case of IDRs. This observation should be systematically investigated in future work using rigorous approaches for the accurate estimation of solvent entropy change.

Overall, our results present a detailed atomistic view of the structure, dynamics and interactions within a model biomolecular condensate, and demonstrate the power of MD simulations in such applications. We hope that our results will stimulate further work on linking the microscopic, physicochemical aspects of disordered protein–protein interactions and condensate formation with their biological roles.

## Methods

All MD simulations and analyses were carried out using the GROMACS 5.1.4 package^98,99^, employing Amber99SB-ILDN force field^95^, TIP4P-D water model^35^, and a modeling protocol described elsewhere^32^. Data processing was carried out using custom scripts in GNU bash 4.3.48 and Python 3.6.1. Data visualization and statistical analyses were carried out in R Studio^100^, using the ggplot2 package^101^ for heatmap visualizations and the PK package^102^ for least-squares fitting of a biexponential model to the tail of the pressure tensor autocorrelation functions.

### Single-copy simulations

The starting models for MD simulations of the C-terminal RGG3 (residues 454–526) of human FUS (UniProt ID: P35637) were derived via RaptorX^103^ and Phyre2^104^ webservers. Simulations were set up in tandem for both acquired models using identical parameters. Single molecules were placed in a cubic box with a side length of 9 nm, followed by 1000 steps of energy minimization in vacuo under 3D periodic boundary conditions (PBC), alternating conjugate-gradient and steepest descent approaches every 100 steps. The simulation boxes were then filled with ~24,000 water molecules, while Na^+^ and Cl^−^ ions were added to a final concentration of 0.1 M at a net system charge of zero (Table 1). Both steps were followed by energy minimization as described above. System equilibration was conducted following a three-step protocol, using one equilibration run in the canonical (NVT) ensemble, with a 0.5 fs time-step for 5000 steps, and two sequential runs in the isothermal-isobaric (NPT) ensemble, with time-steps of 0.5 fs for 25,000 steps and 1 fs for 250,000 steps. A leap-frog algorithm was used for integration with PBC. In both energy minimization and equilibration, neighbor lists were updated every 10 steps, following a Verlet-scheme based grid-search approach. Similarly, in both stages, the van der Waals interactions were treated with a twin-range (1.2/1.6 nm) spherical cutoff function, while electrostatics were treated using the particle-mesh Ewald method with a real space cut-off of 1.2 nm, 0.12 nm grid and cubic interpolation. The bonds involving H atoms were constrained using LINCS^105^. To control temperature at 310 K, Nose–Hoover thermostat^106^ was used with a relaxation time of 0.5 ps for all steps. To keep isotropic pressure constant at 1.013 bar in the second stage, Berendsen barostat^107^ was used, while Parrinello–Rahman barostat^108^ was used in the final step, with the compressibility of 4.5 × 10^−5^ bar^−^and the relaxation time of 10 ps^1^ in both cases. Coupling was done separately for water and protein molecules in all cases. Two 100 ns-long sampling runs, one for each model (RaptorX and Phyre2), using the same parameters and a 2-fs timestep, were set up in order to derive starting structures for multi-copy simulations (see below). Both models converged to a similar radius of gyration (*R*_g_) within 30 ns of simulation. A 1 µs production run, started using the Phyre2-derived model, was carried out using the same parameters as in the last equilibration run, except for an adjustment of the twin-range spherical cut-off (1.0/1.2 nm) for van der Waals interactions. Finally, three 1 µs MD replicas of the single-copy system were obtained using independent equilibration in each case and a simulation protocol as described above (3 µs in total).

### Multi-copy (dense-phase) system preparation

Three cubic simulation boxes with a side length of 15 nm were each filled with 24 copies of FUS RGG3 by randomly selecting structures from a set of structures drawn from the single-molecule sampling runs at regular intervals, to a final concentration of 11 mM (89.2 g l^−1^) in each box. Molecules were placed in a randomized fashion with regards to both molecular identity and orientation on a regularly spaced grid using a custom script, ensuring that steric clashes are avoided.

### Dense-phase simulations

Three independent simulations of the 24-copy systems were obtained following the same energy minimization, equilibration and production run protocols as for the single-copy simulations. The simulation boxes were filled with ~100,000 water molecules, while Na^+^ ions and Cl^-^ ions were added to a final salt concentration of 0.1 M at a net system charge of zero (Table 1). Coordinates and energies were written out every 2 ps with the final simulation time of 1 µs per simulation (3 µs in total). Two of the boxes were additionally simulated for 50 ns in the NVT ensemble for viscosity determination. Energies and coordinates were written out every 10 fs and 10 ps, respectively.

### Water simulations

Pure water was simulated using cubic boxes of different size (edge-lengths of 3, 4, or 5 nm) to determine its in silico rheological properties. All boxes were first energy minimized as described above, followed by the addition of Na^+^ and Cl^−^ ions to a final concentration of 0.1 M. After additional energy minimization, equilibration was conducted as for single-copy protein simulations, followed by 100-ns production runs for each system, using the same parameters as for protein NVT simulations and writing out energies and coordinates every 10 fs and 2 ps, respectively.

### In silico rheology

System shear viscosities and translational diffusion coefficients of solute molecules were estimated following the procedures by von Bülow et al.^83^, relying on the previous work of Hess et al.^109^, who explored ways to determine viscosities from simulations, and Yeh and Hummer et al.^110^, who investigated how the system size influences the diffusion coefficients of simulated molecules. Specifically, shear viscosities were extracted from NVT simulations via the green-Kubo formula:

1 $${\eta }_{{ij}}=\,\frac{V}{{k}_{{{B}}}T}\,\int _{0}^{\infty }{C}_{{ij}}\left(t\right){dt}$$

where *V* denotes the volume of the simulation box, and *C*_ij_ represents the autocorrelation function:

2 $${C}_{{ij}}\left(t\right)=\left\langle {P}_{{ij}}\left(t\right)\,{P}_{{ij}}(0)\right\rangle$$

of the pressure tensor elements ${P}_{{ij}}=\,{P}_{{xy}},\,{P}_{{xz}},\,{P}_{{yz}},\,\frac{{P}_{{xx}}-\,{P}_{{yy}}}{2},\frac{{P}_{{xx}}-\,{P}_{{zz}}}{2},\,{and}\,\frac{{P}_{{yy}}-\,{P}_{{zz}}}{2}$.

The autocorrelation function was numerically integrated between 0 and 1 ps, followed by analytical integration up to infinity. The analytical part of the integral was determined by a least-squares fit of the data between 1 and 4 ps to a bi-exponential function:

3 $${C}_{{ij}}\left(t\right)={a}_{0}{e}^{-\frac{t}{{\tau }_{0}}}+\,{a}_{1}{e}^{-\frac{t}{{\tau }_{1}}}$$

Shear viscosity *η* was then determined by averaging over the *η*_ij_ of the evaluated pressure tensor elements.

Translational diffusion coefficients, *D*_t_, were extracted for individual molecules by evaluating the center-of-mass MSD curves, considering that:

4 $${MSD}=c+6{D}_{{{t}}}^{{PBC}}\tau$$

for *τ* approaching infinity. This equation was fitted in a linear regime between 10 and 30 ns for the 24-copy systems, and between 0 and 5 ns for the single molecule. The constant *c* in the formula accounts for an offset caused by non-diffusive behavior over very short time-separations. As established by Yeh and Hummer et al.^110^, the diffusion coefficients extracted from MD simulations are not directly comparable to experimental data, but need to be corrected for size-dependent effects that arise from PBC. Applying this correction, the diffusion coefficient *D*_t_ can be determined as:

5 $${D}_{{{{\rm{t}}}}}={D}_{{{t}}}^{{PBC}}+\frac{{k}_{{{B}}}T\xi }{6\pi \eta L}$$

where *L* is the length of the simulation box, *η* is the viscosity of the system that the particle is simulated in, and *ξ* = 2.837297, a term arising from the cubic lattice (see Yeh and Hummer et al.^110^ and references therein). Additionally, to enable a more direct comparison with experimental values, Fennel et al.^84^ proposed to multiply the corrected diffusion coefficient by the ratio of the simulated and experimentally determined viscosities:

6 $${D}_{{{{\rm{t}}}}}^{{pred}}={D}_{{{t}}}\cdot \frac{{\eta }_{{sim}}}{{\eta }_{{expt}}}$$

This step should account for inaccuracies in how well the solvent characteristics are reproduced by the water model used. The experimental viscosity at 310 K and 0.1 M salt is *η*_expt_ = 0.694 mPa s^84^.

### Trajectory analysis

System energies, *Rgs* and SASA of individual molecules, and the interatomic distances between and within molecules were analyzed using GROMACS^98^. Energy-related terms were analyzed using an output smallest time-step of 2 ps. A distance of 3.5 Å between centers of any two non-hydrogen atoms was used as a cut-off for defining inter-residue contacts. Instantaneous interaction valency of a protein molecule was defined as the number of partners a given molecule was in contact with at a given time point.

### Cluster analysis

A maximum size of protein-protein interaction clusters in the multi-copy simulated system was identified using hierarchical clustering applied to minimum-distance matrices, which were evaluated from each MD trajectory of the multi-copy system sampled at every 10 ps using GROMACS *mindist*. The clustering was done in MATLAB (R2009) using the function *cluster* with an applied distance cutoff of 3.5 Å.

### Estimation of expected intermolecular interaction frequencies

The expected frequency of intermolecular interactions as a function of the type of residues involved was determined under the assumption that it depends only on the frequency of the respective residue type in the sequence, according to the following formulas:

7 $${{{\mathrm{if}}}}\; {{{\rm{residue}}}}\; {{{\rm{A}}}}\; {{{\rm{is}}}}\; {{{\rm{equal}}}}\; {{{\rm{to}}}}\; {{{\rm{residue}}}}\; {{{\rm{B}}}}\!\!:expected=\frac{{n}_{{{A}}}\cdot \,{n}_{{{B}}}}{{N}^{2}}$$

8 $${{{\mathrm{if}}}}\; {{{\rm{residue}}}}\; {{{\rm{A}}}}\; {{{\rm{is}}}}\; {{{\rm{not}}}}\; {{{\rm{equal}}}}\; {{{\rm{to}}}}\; {{{\rm{residue}}}}\; {{{\rm{B}}}}\!\!:{expected}=\frac{{n}_{{{A}}}\cdot {n}_{{{B}}}\cdot 2}{{N}^{2}}$$

where *n*_A_ is the number of residues of type *A* in the sequence, *n*_B_ is the number of residues of type *B* in the sequence, and *N* is the length of the sequence.

### Identification of interaction motifs

Regions with increased contact formation frequency in simulations were determined by assigning a value of interactivity to each residue as the number of contacts (either inter- or intramolecular, depending on the context) of a given residue with all other residues. The interactivity was normalized by the maximum value in a given set for the purposes of visual representation, followed by smoothing of the resulting interactivity-profiles using the Savitzky–Golay filter:

9 $${y}_{{{t}}}=\frac{1}{h}\left({\sum }_{i\,=\,-\,\frac{w-1}{2}}^{\frac{w-1}{2}}{a}_{{{i}}}\,{x}_{{{t}}+{{i}}}\right)$$

Normalization parameters *h* and coefficients *a*_i_ for window sizes *w* of 5, 7, and 9 were used as suggested by Lohninger^111^. Identification of interaction motifs as the minima in the second derivative of the interaction profiles was also performed via the Savitzky–Golay filter. The minima were selected by rejecting all values that were closer than one standard deviation to the mean of the second numerical derivative of the interaction profile. Shapiro–Wilk test was used to ensure distribution normality. This process was adopted for *w* = 5, 7, and 9, and each minimum was assigned as the middle point of a putative interaction motif of a window-size w. The final assignment of interaction motifs considered all stretches along the sequence that were found to include a putative interaction motif in at least two different window sizes. Motifs were defined at full or reduced certainty, depending on whether a residue was identified by all three or just two of the smoothing window sizes, respectively.

### Error propagation

Error propagation was carried out using the following formula^112^:

10 $${\varepsilon }_{{prop}}=\sqrt{{\sum }_{i=1}^{n}{\left(\frac{\partial f}{{\partial x}_{{{{\rm{i}}}}}}{\varepsilon }_{{{{\rm{i}}}}}\right)}^{2}}$$

Here, *ε*_prop_ denotes the propagated error, whereas *ε*_i_ denotes the error of individual values related by a function f(*x*_1_, *x*_2_ … *x*_n_).

### Entropy calculation

The configurational entropy was estimated using the maximum information spanning tree (MIST) approximation^113^ as implemented in the PARENT program suite^30^ and also described elsewhere^32^. Here, all MD trajectories were converted to bond-angle-torsion coordinates. The convergence of configurational entropy (*S*_conf_) was assessed for single-copy systems using cumulative plots with a 50-ns time step. To account for a relatively slow convergence (Supplementary Fig. 1C), entropy calculations were carried out for the entire 1-µs trajectories. The relative entropies (Δ*S*_conf_) of a protein in single-copy and multi-copy systems were averaged over all possible combinations of the 3 single-protein copies and the 24 copies in the dense-phase systems. The final values of Δ*S*_conf_ were multiplied by the temperature (*T* = 310 K) and converted to kcal mol^−1^ units.

### RDF calculations

A RDF for water molecules with respect to the protein was calculated for the complete 1-µs trajectories of the dense-phase and single-copy (Supplementary Fig. 1E) systems using a 100 ps timestep. From the cumulative RDFs, a distance-dependent variation in the number of water molecules was estimated between the independent runs (Supplementary Fig. 1E, inset) and a characteristic distance of 1.25 nm (the inflection point) was identified as the boundary of the bound water shell. All further estimates of change in the number of water molecules between the dense and the single-copy systems were thus made for a water shell with the effective radius of 1.25 nm. Also, for the dense-phase and single-copy systems, RDFs were calculated for 0–1 and 999–1000 ns ranges of MD trajectories using 10 and 5 ps timesteps, respectively. For multi-copy systems, RDFs were calculated for each protein copy independently. RDF calculations were performed using the GROMACS *rdf* utility with the fixed maximum distance from protein atoms of 5 nm.

### Conformational statistics

Conformational dynamics of RGG3 was analyzed using a sliding-window scheme of overlapping pentamer (5-mer) and decamer (10-mer) peptide fragments with single-residue window shifts. For each of the thus defined fragments, all of their local conformations in the multi-copy simulations were combined into a single trajectory (24  µs/1 ns time step). Pentamer and decamer trajectories were constructed for each multi-copy replica and later combined into a single, master trajectory (72 µs/3 ns time step).

### Conformational clustering

Conformational clustering on the subsampled pentamer and decamer ensembles was performed using the GROMACS *cluster* utility with backbone RMSD cut-offs of 0.05 and 0.1 nm defining the cluster perimeter for pentamers and decamers, respectively. For each peptide fragment, the most populated conformational clusters were identified using combined MD trajectories (also for each independent replica).

### RMSF calculations

Combined pentamer and decamer trajectories were used to calculate backbone atom-positional root-mean-fluctuations (RMSF) for each overlapping peptide fragment using the GROMACS *rmsf* utility. The RMSF value of a middle residue in the analyzed peptide fragments (3rd in pentamers and 5th in decamers) was used to reconstruct the RMSF profile for the full-length protein.

### Time-resolved contacts statistics

Estimation of residual contacts lifetimes was performed for the dense-phase systems using a high-performance *Python* framework specially developed for this purpose (*MD_contacts*, https://github.com/Benjabern/MD_contacts, *in preparation*). Intermolecular contacts were collected over the last 0.4 µs of MD trajectories for the dense-phase systems using a 4-ps timestep (10^5^ frames in total), and for each residue-residue contact, an average uninterrupted interval, over which it was present, was calculated. Statistics for the above-defined interaction motifs (stickers, 5 in total) and inter regions between them (spacers, 6 in total) were analyzed for each protein copy individually and averaged between them. For the analysis of individual protein statistics, the sticker ranges in RGG3 sequence were fixed as follows: 8–17 (st1), 24–33 (st2), 37–43 (st3), 48–54 (st4), 64–70 (st5).

### Application of the fractal model

To model and visualize the topology of the self-assembled RGG3 clusters, a previously developed analytical framework assuming the fractal mode of protein self-association^32^ was employed. According to this model, condensate architecture across length scales can be described as a function of protein valency (*n*) and compactness, or volume fraction (*ϕ*). These two parameters allow a direct assessment of the topological organization of self-assembled protein clusters across scales via the calculation of a fractal dimension (*d*_F_) as follows:

11 $${d}_{{{F}}}=\frac{3}{1-\frac{\log \varphi }{\log \left(n+1\right)}}$$

The valency (or the coordination number, *n)* can be directly obtained from the analysis of protein–protein contacts (see above), while compactness (*ϕ)* is defined as the ratio between the molecular (van-der-Waals) volume (*V*_mol_) and the apparent one, as derived from the *R*_g_:

12 $$\varphi =\frac{{V}_{{mol}}}{\frac{4}{3}\pi {R}_{g}^{3}}$$

where *V*_mol_ is proportional to the molecular weight (*M*_w_) with the used prefactor *k* as 1.21^32^:

13 $${V}_{{mol}}=\kappa \,* \,{M}_{{{w}}}$$

*d*_F_, *R*_g_, and *n* were estimated from independent MD trajectories of the multi-copy system and also averaged over the last 0.3 µs. These average parameters were further used to generate a visual representation of the corresponding protein clusters (condensates) using the FracVAL algorithm^114^, as described elsewhere^32^. The clusters generated by FracVAL for visualization purposes contained 1024 proteins.

## Supplementary information

A Combined Supplementary Information PDF

Description of Additional Supplementary Files

Supplementary Movie 1

Supplementary Data 1

## Data Availability

The authors declare that the data supporting the findings of this study are available within the paper and its Supplementary Information files. A combined Supplementary Information PDF includes Supplementary Figs. 1–3 and Supplementary Table 1. Supplementary Movie 1, initial and final conformations from MD simulations (Supplementary Data 1) are available as Supplementary Data.
