# Supplementary material for: Dynamic yet well-defined organization of the FUS RGG3 dense phase
Source: Commun Chem. 2026 Mar 21;9:177. doi: 10.1038/s42004-026-01974-z (PMC13172571; doi:10.1038/s42004-026-01974-z)
Supplement: Supplementary file 1 — A Combined Supplementary Information PDF [file 42004_2026_1974_MOESM1_ESM.pdf]

Supplementary Information for

Dynamic yet well-defined organization of the FUS RGG3 dense phase

Anton A. Polyansky[1,2#*], Benjamin Frühbauer[3,4#] & Bojan Žagrović[1,2*]

[1]Max Perutz Labs, Vienna Biocenter Campus (VBC), Campus Vienna Biocenter 5, A-1030, Vienna, Austria

[2]University of Vienna, Campus Vienna Biocenter 5, A-1030, Vienna, Austria

[3]ETH Zurich, Institute of Biochemistry, Otto-Stern-Weg 3, CH-8093, Zurich, Switzerland

[4]ETH Zurich, Bringing Materials to Life Initiative, Otto-Stern-Weg 3, CH-8093, Zurich, Switzerland

[#]These authors contributed equally

[*]e-mail: anton.polyansky@univie.ac.at, bojan.zagrovic@univie.ac.at

This PDF includes:

Supplementary Figure 1-3
Supplementary Table 1

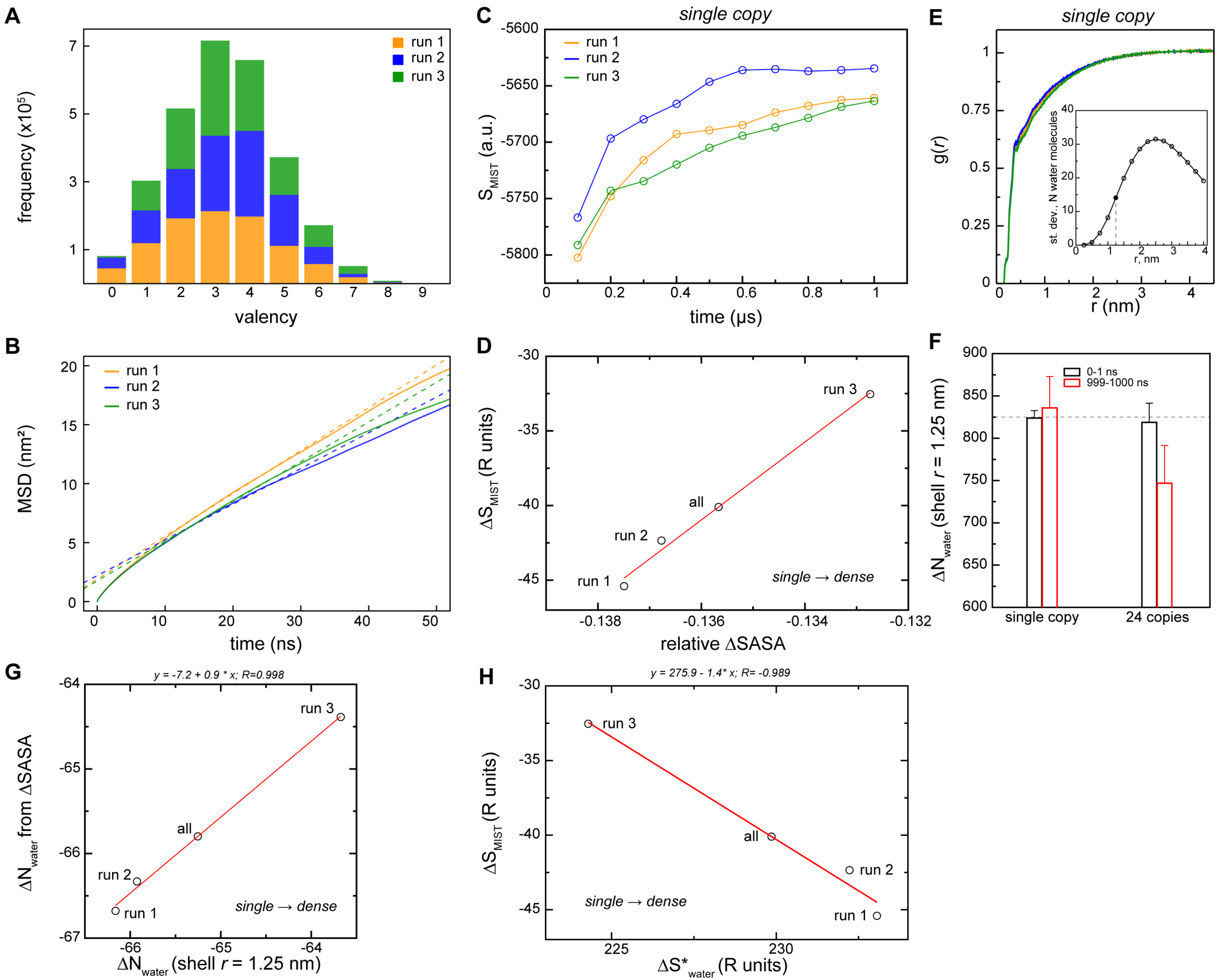
A
frequency (x10⁵)
valency
run 1
run 2
run 3
B
MSD (nm²)
time (ns)
run 1
run 2
run 3
C
single copy
S_MIST (a.u.)
time (μs)
run 1
run 2
run 3
D
ΔS_MIST (R units)
relative ΔSASA
run 1
run 2
run 3
all
single → dense
E
single copy
g(r)
r (nm)
st. dev., N water molecules
r, nm
F
ΔN_water (shell r = 1.25 nm)
0-1 ns
999-1000 ns
single copy
24 copies
G
y = -7.2 + 0.9 * x; R=0.998
ΔN_water from ΔSASA
ΔN_water (shell r = 1.25 nm)
run 1
run 2
run 3
all
single → dense
H
y = 275.9 - 1.4* x; R= -0.989
ΔS_MIST (R units)
ΔS*_water (R units)
run 1
run 2
run 3
all
single → dense

Supplementary Figure 1. **A.** Distribution of average valencies over all 24 RGG3 copies over the last 0.4 μs of each independent MD simulations of the dense phase. **B.** MSD-curve fitting for estimation of diffusion coefficients in the RGG3 dense phase with linear regime between 10-30 ns. MSD curves were calculated using complete 1 μs MD trajectories. **C.** Convergence of RGG3 configurational entropy (S_{MIST}) in the single-molecule context. Cumulative plots were generated using MIST approximation (see Methods) and a 100-ns time step. **D.** Linear regression between configurational entropy changes and the corresponding changes in SASA of RGG3 upon transitioning into the dense phase. Relative entropy and SASA values were obtained using the complete 1-μs MD trajectories. **E.** Radial distribution functions (RDFs) for water molecules with respect to the protein in the three single-copy systems calculated for the complete 1-μs MD trajectories. *Inset*: A distance-dependent variation in the number of water molecules between the independent runs estimated from cumulative RDFs. The inflection point corresponds to a characteristic distance of 1.25 nm (shown with a dashed line). **F.** The number of bound shell water molecules in the first and in the last ns of MD trajectories as estimated from the corresponding RDFs. **G.** Linear regression between the difference in the number of bound-shell water molecules as estimated from RDFs obtained in the dense and the single-copy systems and the respective changes in SASA. **H.** Linear regression between RGG3 configurational entropy changes and the corresponding changes in solvent entropy upon transitioning of the protein into the dense phase.

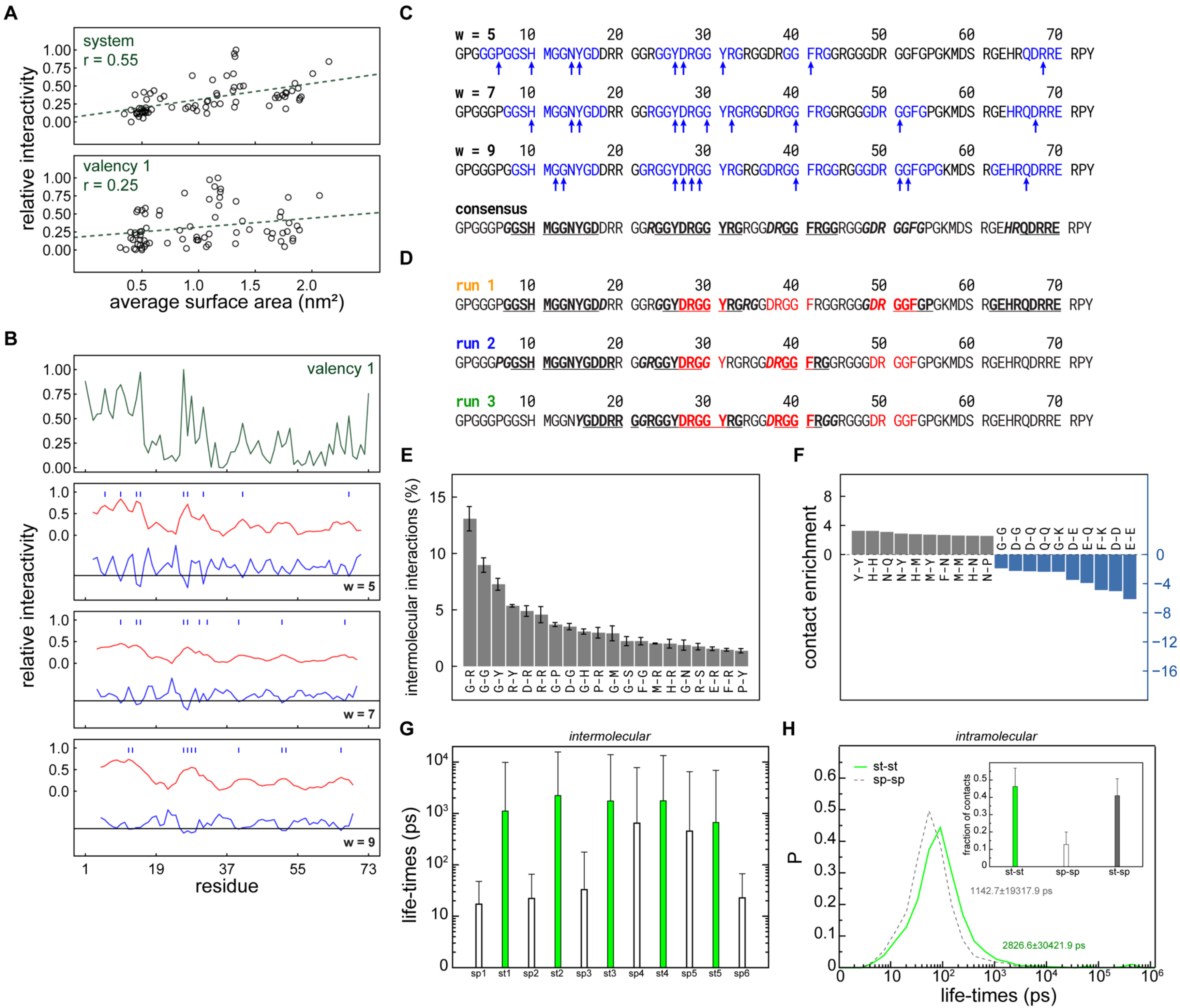
A
system
r = 0.55
valency 1
r = 0.25
relative interactivity
average surface area (nm²)
B
valency 1
relative interactivity
w = 5
w = 7
w = 9
residue
C
w = 5
GPGGGPGGSH MGGNYGDDRR GGRGGYDRGG YRGRGGDRGG FRGGRGGGDR GGFGPGKMDS RGEHRQDRRE RPY
w = 7
GPGGGPGGSH MGGNYGDDRR GGRGGYDRGG YRGRGGDRGG FRGGRGGGDR GGFGPGKMDS RGEHRQDRRE RPY
w = 9
GPGGGPGGSH MGGNYGDDRR GGRGGYDRGG YRGRGGDRGG FRGGRGGGDR GGFGPGKMDS RGEHRQDRRE RPY
consensus
GPGGGPGGSH MGGNYGDDRR GGRGGYDRGG YRGRGGDRGG FRGGRGGGDR GGFGPGKMDS RGEHRQDRRE RPY
D
run 1
GPGGGPGGSH MGGNYGDDRR GGRGGYDRGG YRGRGGDRGG FRGGRGGGDR GGFGPGKMDS RGEHRQDRRE RPY
run 2
GPGGGPGGSH MGGNYGDDRR GGRGGYDRGG YRGRGGDRGG FRGGRGGGDR GGFGPGKMDS RGEHRQDRRE RPY
run 3
GPGGGPGGSH MGGNYGDDRR GGRGGYDRGG YRGRGGDRGG FRGGRGGGDR GGFGPGKMDS RGEHRQDRRE RPY
E
intermolecular interactions (%)
G-R G-G G-Y R-Y D-R R-R G-P D-G G-H P-R G-M G-S F-G M-R H-R G-N R-S E-R F-R P-Y
F
contact enrichment
contact depletion
Y-Y H-H N-Q N-Y H-M M-Y F-N M-M H-N N-P
G-G D-G D-Q Q-Q G-K D-E E-Q F-K D-D E-E
G
intermolecular
life-times (ps)
sp1 st1 sp2 st2 sp3 st3 sp4 st4 sp5 st5 sp6
H
intramolecular
st-st
sp-sp
fraction of contacts
st-st sp-sp st-sp
1142.7±19317.9 ps
2826.6±30421.9 ps
P
life-times (ps)

Supplementary Figure 2. A. Relative interactivity at all valencies and valency 1 of different residues as a function of their average surface area over the last 0.4 µs of MD trajectories of the dense phase with the corresponding Pearson's R. **B.** Visual example of peak picking using the Savitzky-Golay filter: The interactivity profile (top) is smoothened (red) and the second derivative (blue) is numerically determined using different window size, as indicated. The black lines indicate the distance of one standard deviation from the arithmetic mean of the second numerical derivatives, which is used as a cutoff for peak-picking. Blue bars above the smoothened interactivity profiles (red) indicate identified interactivity peaks. **C.** Sequence representation of Savitzky-Golay-identified interactivity peaks at different window sizes; arrows indicate the positions identified as peaks in the profile, while colored regions include residues that contributed to the peak signal. The bottom sequence shows the identified stickers in bold and underlined or bold and italic for full or reduced confidence, respectively. **D.** Comparison of stickers between replica simulations. **E.** The twenty most frequently occurring types of pairwise intermolecular contacts and the associated frequencies in percent. Error bars indicate standard deviation between replicate trajectories. **F.** The ten most enriched and depleted interactions over all valencies. **G.** Average life-times for intermolecular contacts in different sticker and spacer regions of RGG3 sequence. **H.** Life-times of intramolecular contacts between interaction stickers and spacers in the RGG3 protein. The distributions comprise all individual values of life-times for selected residual contact types obtained over the last 0.4 µs of three independent runs of the 24-protein-copy system. *Inset:* Average fractions of all intramolecular contacts distributed between sticker and spacers regions. Error bars depict standard deviations.

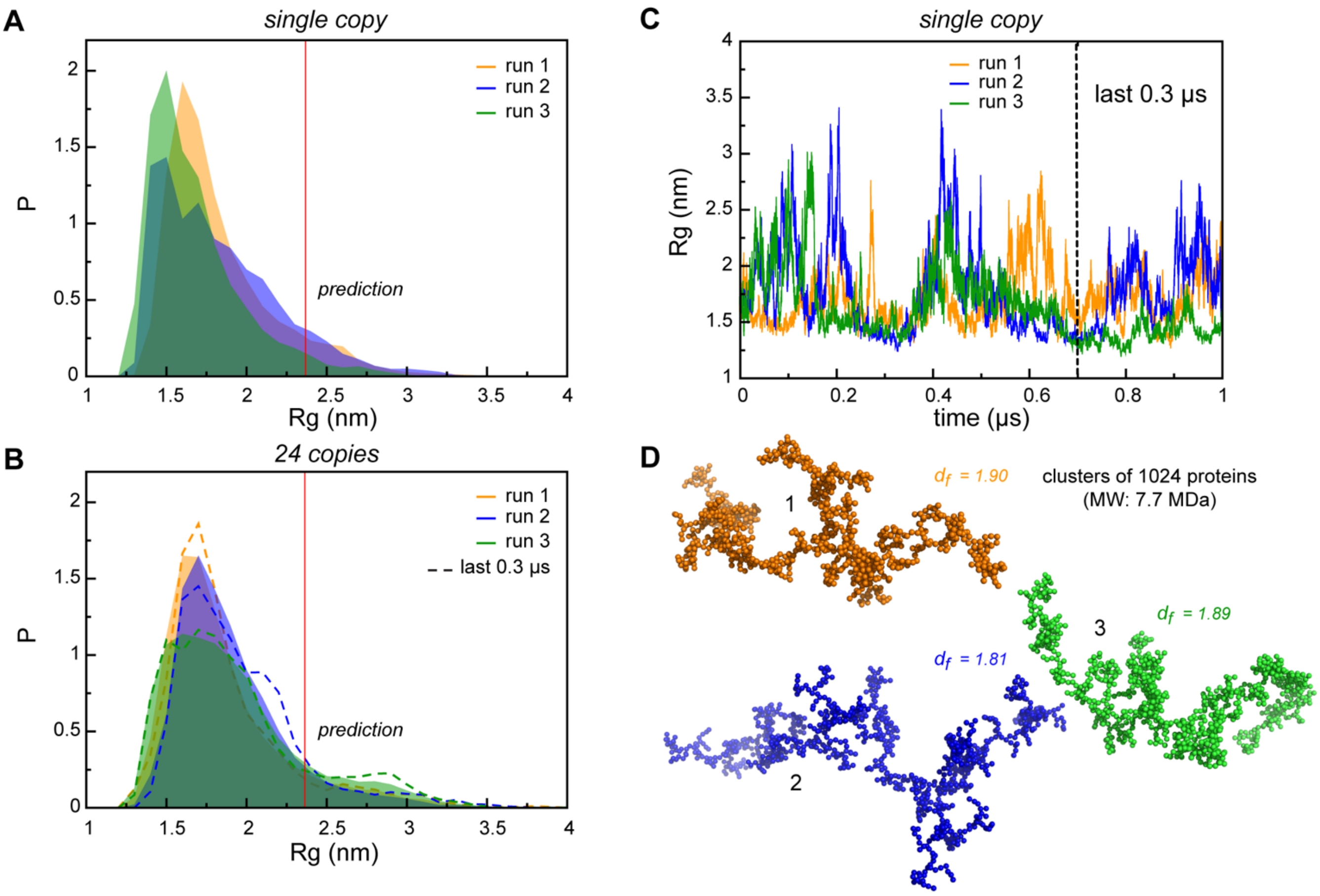

Supplementary Figure 3. Distributions of radii of gyration (*Rg*) RGG3 in (**A**) single-copy and (**B**) multi-copy systems. Complete MD trajectories were used to collect *Rg* statistics. *Rg* distributions in the dense phase over the last 0.3 µs of MD trajectories are shown with dashed lines. Theoretical Rg_{rc} value for a 72-aa disordered protein chain (see Methods) is shown with a vertical red dashed line. **C.** Time evolution of *Rg* in RGG3 single-copy MD simulations. **D.** Representative coarse-grained 1024 particle clusters obtained by FracVAL algorithm (see Methods). The clusters were reconstructed using the d_F and the averaged *Rg* values over the last 0.3 µs of each independent MD simulations of the dense phase. Fractal dimensions (d_F) corresponding to each cluster are indicated.

Supplementary Table 1 Shear viscosity values obtained in different MD systems

system*	concentration, g · l^{-1}	viscosity, mPa · s	± st. dev.
TIP4PD	0	0.82	0.01
single copy (run1)	17	0.90	0.04
24 copies (run1)	89	1.22	0.04
24 copies (run3)	89	1.21	0.05
24 copies (average)	89	1.22	0.03

* NVT simulations, see Methods for the details
