# Supplementary material for: Dynamic yet well-defined organization of the FUS RGG3 dense phase
Source: Commun Chem. 2026 Mar 21;9:177. doi: 10.1038/s42004-026-01974-z (PMC13172571; doi:10.1038/s42004-026-01974-z)
Supplement: Supplementary file 2 — Description of Additional Supplementary Files [file 42004_2026_1974_MOESM2_ESM.pdf]

Description of Additional Supplementary Files:

File: Supplementary Data 1

Description: **Configurations of MD systems**.

Initial and final configurations for all MD systems used in the study.

File: Supplementary Movie 1

Description: FUS RGG3 self-associate in the dense phase.

All-atom, explicit-solvent MD simulation with 24 copies of FUS RGG3, corresponding to the concentration of 89 mg/ml (water and ions are not shown for clarity). The movie shows the complete 1 μs of the simulated trajectory with a 1-ns step (movie rate: 25 ns of MD in 1 s). Proteins are shown in cartoon representation. Individual protein copies are highlighted in shades of green, while the periodic images of the central simulation box in x-, y-, and z directions, which are used to treat the finite size effects in MD simulations, are shown in gray. Note that the periodic images exhibit identical movements as the molecules in the central box.
